# The reproducibility crisis in the age of digital medicine

**DOI:** 10.1038/s41746-019-0079-z

**Published:** 2019-01-29

**Authors:** Aaron Stupple, David Singerman, Leo Anthony Celi

**Affiliations:** 1 Baystate Medical Center, Springfield, MA USA; 2 Beth Israel Deaconess Medical Center, Boston, MA USA; 3 University of Virginia, Charlottesville, VA USA; 4 Massachusetts Institute of Technology, Cambridge, MA USA

**Keywords:** Preclinical research, Outcomes research

## Introduction

If anyone doubts the explosive growth of interest in digital medicine, consider a recent conference and workshop in Beijing, jointly organized by the People’s Liberation Army General Hospital and MIT Critical Data to showcase the opportunities and challenges of applying machine learning to the kind of data routinely collected during the provision of care.^1^ In person, 500 attendees heard a keynote and panels and participated in a health data hackathon. Online, however, the event was streamed to more than one million unique viewers.^2^

As databases of medical information are growing, the cost of analyzing data is falling, and computer scientists, engineers, and investment are flooding into the field, digital medicine is subject to increasingly hyperbolic claims. Every week brings news of advances: superior algorithms that can predict clinical events and disease trajectory, classify images better than humans, translate clinical texts, and generate sensational discoveries around new risk factors and treatment effects. Yet the excitement about digital medicine—along with the technologies like the ones that enable a million people to watch a major event—poses risks for its robustness. How many of those new findings, in other words, are likely to be reproducible?

Digital medicine must take steps to avoid a reproducibility “crisis” of the kind that has engulfed other areas of biomedicine and human science in the last decade and shaken public confidence in the validity of scientific work. The goal of this paper is to use a historical perspective on reproducibility and its current crisis to suggest how digital medicine can avoid a reproducibility crisis of its own.

## Interpreting the reproducibility crisis

Researchers in many fields now widely accept the existence of a “replication crisis” or “reproducibility crisis.” For our purposes here, we take reproducibility to mean “obtaining the same results from the conduct of an independent study whose procedures are as closely matched to the original experiment as possible” (also known as “research reproducibility” or simply “replicability”).^3^ A sense of crisis itself began with the widespread awareness of reproducibility failures among the public, when the Center for Open Science announced in 2015 that it could confirm just 39 of 100 published studies in psychology.^4^ For many scientists, however—not just in psychology—the Center’s Reproducibility Project merely publicized their existing fears that unverifiable results were passing science’s institutional checks and becoming accepted as findings and entrenched as facts. What is important to note is that the sense of urgency is not confined to the supposedly “softer” psychology or social sciences. In fact, a 2016 *Nature* survey found that doctors and others in biomedicine are the most concerned of all.^5^

Much of the criticism and comment about reproducibility and solutions to the crisis—both real and perceived—focuses on statistics and methodology. In the past decade, statisticians have shown how statistics may be unintentionally misused or, in some cases, intentionally abused as researchers try to produce results that appeal to professional colleagues and attract potential funders.^6–8^ Commonly proposed solutions include better statistical literacy and behavioral norms, reform of peer reviewed journals, and institutional realignment of rigorous science.^9^ Other critiques have focused on the peer review process as a contributor to the crisis.

In the next sections, we draw on the history of science to argue that the current emphases on statistics and peer review must be understood in the context of an essential and longstanding tension in science, between innovation and reliability. Without addressing the way science’s contemporary institutions and structures influence this tension to create conditions for a reproducibility crisis, neither the perception of crisis nor the problem itself is likely to get better.

## The changing meaning of reproducibility

By many accounts, digital medicine holds the potential to transform how scientists and physicians study human health. It is worth considering, therefore, a few other moments when novel technologies reshaped the scientific enterprise, and the effects those technologies had on what we might today call reproducibility.

During one of the key episodes in the creation of modern science, Robert Boyle and the Royal Society proposed an entirely new model of learning things about the world. Rather than deduce facts like philosophers, experimenters would achieve consensus about nature through observation. As today, Boyle’s innovation was in new ways of sharing his data. By circulating reports that described his methods in exhaustive detail, his radical “literary technology” of “virtual witnessing” persuaded readers of his findings without those readers ever having to make comparable tests themselves. In fact, those who tried to actually produce Boyle’s results solely from those reports failed. As the historians Simon Schaffer and Steven Shapin demonstrated, no one could redo Boyle’s “trials” without direct assistance from someone who had witnessed the original experiment directly rather than virtually.^10^

If reproducibility in the age of digital medicine now means the practical (rather than hypothetical) ability to redo an experiment and obtain the same finding, that is partly because the cost of such recreations has plummeted. In most fields and until very recently reproductions were implausibly costly. When the historian Otto Sibum, in the 1990s, tried to re-create James Joule’s landmark 1840 experiments on the mechanical equivalent of heat, Sibum found that Joule possessed a whole set of skills and tools that would have taken anyone else a lifetime and fortune to acquire. Likewise, Louis Pasteur shrewdly exploited the difference between science in public and private. In large and highly publicized experiments, Pasteur famously demonstrated his anthrax vaccine on farm animals in northern France and preached the importance of his rational method, but his notebooks reveal that he dissimulated about his laboratory’s actual procedures and routinely stretched truth and ethics in pursuit of recognition.^11^

## Peer review would not protect us

Today, it is peer review that supposedly guarantees that published findings are correct—and implicitly, that such findings could be reproduced if other researchers tried.^12^ But as several scholars have shown, the current incarnation of peer review—in which submissions to journals are refereed by two anonymous colleagues—is a historical accident, far from a procedure designed to separate truth from fiction.

There have been various gatekeepers to publication since the seventeenth century, and most often these gatekeepers are editors rather than peer scientists. Additionally, the gatekeeping mechanisms have served multiple social, political, and institutional aims beyond simply adjudicating the correctness of findings. Alex Csiszar^13^ shows an early case in which these incentives ran afoul of each other. When philosopher William Whewell and astronomer John Lubbock disagreed about an 1834 study on the orbit of Venus, Whewell’s admiring view was made public and Lubbock’s suppressed, despite the latter’s concern about “grievous errors.” Aileen Fyfe^14^ writes that the French Academie had even tried assigning committees to replicate the experiments in submitted papers, a costly project that was abandoned. In choosing not to follow the Academie’s lead, the Royal Society disclaimed liability for the “certainty of the facts” contained in the pages of its *Transactions*. Instead their selection committee had prioritized “singularity.”

Only in the post-World War II period did something like peer review as we know it today become standard practice. Once again, the system was not carefully constructed as a method for assessing truth. Instead, it evolved alongside the postwar military–industrial–scientific complex’s need to distribute research funds. Peer review was a compromise between scientists’ desire for independent control and the need for democratic accountability.^15^

As peer review became the determinant of funding, promotion, and publicity, some critics, like Paul Meehl, warned that a “zealous and clever investigator” could engage in a years-long research program that was never corroborated or refuted. Partly this was because, as National Science Foundation Director Walter E. Massey noted in 1991, the specialization of research left the scientific community increasingly “vulnerable to falsehoods.” For hundreds of years, however, critics have always pointed to a more subtle conflict: that between the desire to quickly disseminate new scientific findings and the need to ensure that those findings have actually found something. It is no surprise that an increasingly competitive and individualized scientific profession, and growing avenues for publicity and recognition, has ratcheted that conflict even higher.

By the late 1990s, peer review came under heavy criticism. Its many failings, which contribute directly to difficulties with reproducibility, have become well known but bear repeating. Studies with negative or null findings are rarely submitted, let alone published, thereby opening the door to false-positive findings. The total pool of available reviewers is shrinking compared to the growth of submissions. Often only two reviewers analyze a paper, and they do so voluntarily, with little quality control, transparency, or incentives aligned toward thoroughness. Data and code are often unavailable for validation. Reviewers are often not blinded to the authors’ identity, and authors can request specific reviewers, so reputation and connections can bias the critique. Finally, since reviewers are often senior researchers, authors are incentivized to submit work that, while attention-grabbing, does not fundamentally challenge the status quo, however faulty it may be. All these create opportunities for shoddy work to find publication.^16^

## Reproducibility and digital medicine

Reproducibility, for most of the history of modern science, has arguably been more hypothetical than real. In practice, re-creating Boyle’s or Joule’s experiments required an implausibly specific and costly set of techniques. The Reproducibility Project’s ongoing effort to stress-test hundreds of psychology experiments is difficult and expensive. But as more data and computing power become cheaply available to the field of digital medicine, actually doing an analysis over again has become far more realistic. This means that standards for what counts as “reproducible research” may also be changing. Digital medicine findings may have to be more robust because they will be subjected to more, and more intensive, scrutiny than other scientific findings have historically been—and they will be subjected to that scrutiny sooner.

New initiatives are collecting and integrating health-related data at an unprecedented scale. The National Institute of Health’s All of Us Research Program,^17^ for instance, aims to gather long-term data from one million United States residents to learn more about how lifestyle, environment, and biology can influence health and disease. Meanwhile, projects like the eMERGE national network (supported by the National Human Genome Research Institute)^18^ and the Biomedical Data Translator program (of the National Center for Advancing Translational Sciences)^19^ are bridging divides between data types, such as DNA bio-repositories and electronic health record systems, that have conventionally remained separate. These offer the possibility for large-scale, high-throughput genetic and health research.

All these new resources, coupled to the excitement around deep learning and artificial intelligence, mean that we will soon be drowning in publications touting new biomarkers and breakthrough cures, automated diagnoses and personalized treatments, more accurate prediction and classification algorithms. The time for digital medicine to think about how to promote robust findings and reject irreproducible ones is now—not after a crisis threatens its credibility. Rather than simply forestalling a crisis, however, this necessity ought to be viewed as an opportunity. As an embryonic discipline, digital medicine has the chance to inculcate among its practitioners a healthier set of attitudes towards replication.

## Conclusion: reproducibility and the social order of digital medicine

For digital medicine, it is especially critical to avoid drawing unsubstantiated conclusions from work that appears to rest firmly on impressive gobs of data. Clinical data are a particularly fragile substrate in the sense that it is prone to unique problems ranging from faulty or missing human entry to artifacts and errors that occur in the use of technology for diagnosis and monitoring. The heterogeneity of the global population of diseased humans is also a formidable challenge for mathematical modeling in terms of capturing the variety of biological, environmental, and behavioral confounders that must be measured and accounted for. Finally, the construction of any artificial intelligence based on these data must be as free as possible from the conscious and unconscious bias of those involved in the development of the algorithms.

Digital medicine needs to incentivize good research practices, for instance through pre-publication review, registration of analyses in advance, commitments to publish, or open registries of methods and results. Standards of data and code sharing must transition from mere publication policies to actually allow third parties to reproduce studies.^20^ Emerging guidelines for reporting and evaluating results, such as those for causal inference and reinforcement learning studies, must be refined and expanded across the field.^21,22^ The research economy cannot turn all its incentives toward novelty, emphasize sensational results to generate attention and prestige outside of academic channels,^23^ or overproduce graduates who have to claw for funding and employment. The health of the research environment will directly affect digital medicine’s ability to improve the health of human beings.

At the same time, to guarantee that every result is completely reproducible would stifle the novelty and creativity that are essential for taking advantage of what digital medicine really does offer. As Nosek et al. write, “A healthy discipline will have many false starts as it confronts the limits of present understanding.”^4^ What is important is to make sure that such false starts emerge from a system that takes reproducibility seriously and incorporates voices who know how well-intentioned scientists can be led astray. Fortunately, the inherent openness and accessibility of work in digital medicine, and the relatively low barriers to replication, present an opportunity for the field. Done right, reproducibility should not be a crisis for digital medicine, but rather one of its strengths.
