# Author Correction: The reproducibility crisis in the age of digital medicine

**DOI:** 10.1038/s41746-019-0098-9

**Published:** 2019-03-19

**Authors:** Aaron Stupple, David Singerman, Leo Anthony Celi

**Affiliations:** 1 Baystate Medical Center, Springfield, MA USA; 2 Beth Israel Deaconess Medical Center, Boston, MA USA; 3 University of Virginia, Charlottesville, VA USA; 4 Massachusetts Institute of Technology, Cambridge, MA USA

**Keywords:** Preclinical research, Outcomes research

**Correction to:** *npj Digital Medicine* 10.1038/s41746-019-0079-z, Published online 29 January 2019

In the original version of the published Article, there was a sentence fragment prior to the last sentence under the subheading “Reproducibility and digital medicine” which stated “Some journals, such as PLOS ONE, have made an early”. To improve clarity, the sentence fragment has been removed. This has been corrected in the HTML and PDF version of the Article.

